# Associations between workplace aggression and subsequent mental distress and sick leave among home care workers

**DOI:** 10.1007/s00420-025-02183-2

**Published:** 2025-11-13

**Authors:** Rigmor Harang Knutsen, Morten Birkeland Nielsen, Knut Inge Fostervold, Håkon A. Johannessen

**Affiliations:** 1 Research Group for Work Psychology and Physiology, National Institute for Occupational Health, Gydas vei 8, 0363 Oslo, Norway; 2 Department of Psychology, University of Oslo, Oslo, Norway; 3 Department of Psychosocial Science, University of Bergen, Bergen, Norway

**Keywords:** Workplace mistreatment, Violence, Bullying, Absenteeism, Psychological distress

## Abstract

**Objective:**

The Norwegian home care sector faces staff shortages and high sick leave due to common mental disorders, often linked to work-related stress. This study examined associations between workplace aggression (threats/violence, bullying, and unwanted sexual attention) and subsequent mental distress and sick leave among home care workers.

**Methods:**

A total of 1426 employees (baseline n = 2591) from 130 randomly selected home care services completed surveys on workplace aggression and mental distress (HSCL-5 > 2) at baseline, 8 months, and 14 months. Registry data on medically certified sick leave (with diagnostic codes) were obtained for 1819 participants over 26 months. Mixed-effects lagged logistic regression estimated odds of mental distress, and negative binomial regression calculated incidence risk ratios (IRRs) for sick leave due to mental disorders.

**Results:**

All types of workplace aggression were associated with increased risk of clinically relevant mental distress. Only colleague-perpetrated bullying significantly predicted sick leave for mental disorders (IRR 1.62, 95% CI 1.17–2.23).

**Conclusion:**

Workplace aggression was common and associated with poorer mental health and increased absenteeism. Targeted, multicomponent interventions are needed to prevent aggression and reduce its mental health impact.

## Introduction

Maintaining a stable workforce is a consistent challenge for the home care sector. Sick leave levels are near twice as high as in the general workforce (National Occupational Health Surveillance 2022) and the sector struggles with staff shortages, recruitment, and retainment of nurses (Gautun 2021; Vabo 2012). To secure employee retention in home care, it is necessary to identify and address the antecedents of ill health and absence.

While there are many work-related risk factors that influence the health and well-being of healthcare workers, workers are especially at risk for frequently experiencing physical and psychological workplace aggression (Goh et al. 2022; Liu et al. 2019). The term *workplace aggression* refers to the experience of mistreatment from others that is (a) potentially harmful for an individual, (b) that the target is motivated to avoid, and (c) that occurs while the target is working (Schat and Frone 2011). Two distinct second order categories of workplace aggression are physical violence and psychological aggression. The former is characterized by a physical act typically causing immediate physical harm, e.g. beating, slapping, kicking, or armed assault, whereas the latter involves behaviour of a verbal or symbolic nature that mainly causes psychological harm such as anxiety, e.g. threats, being shouted at, humiliation, and bullying.

Due to the nature and content of home care work, exposure to workplace aggression may be especially detrimental for mental health and work attendance. Workers mainly work alone and must regularly enter the homes of patients they perceive as frightening or dangerous (Hertzberg et al. 2024), while having limited access to immediate support from colleagues, which leaves workers more vulnerable to threatening situations (McPhaul and Lipscomb 2004). However, as previous research on workplace aggression toward healthcare workers have predominantly focused on physicians or nurses working in hospitals, psychiatric or emergency departments, or long-term care facilities (Goh et al. 2022; Lanctôt and Guay 2014; Mento 2020; Spector et al. 2014), there is a shortage of knowledge about how exposure to workplace aggression is associated with mental health and the risk of sick leave for home care workers specifically. The overarching aim of the current study was therefore to examine exposure to different forms of workplace aggression as risk factors for clinically relevant levels of mental distress and sick leave due to common mental disorders among Norwegian home care workers. The study will use *physical violence* from clients as an indicator of physical aggression, whereas *threats of violence from clients*, *unwanted sexual attention*, and *colleague- and supervisor-perpetrated bullying* are used as indicators of psychological aggression. Bullying are situations where an employee experience systematic exposure to psychological aggression from one or more colleagues while simultaneously being unable to defend themselves from this mistreatment (Einarsen 2005).

Although estimates vary, evidence suggests that exposure to physical and psychological violence is prevalent in home care work. A meta-analysis estimated that about 10% of home care workers experienced patient-perpetrated physical violence across a one-year time frame, whereas 36% experienced psychological violence (Byon et al. 2020). In comparison, prevalences rates were 14% for physical and 52% for psychological violence toward professionals such as nurses and physical therapists. A Danish study of aggression toward eldercare workers found rates of 11.9% for bullying, 19.9% for violence, 33.0% for threats, and 9.3% for unwanted sexual attention (Clausen et al. 2012). A review of bullying among colleagues in the nursing profession in general established prevalence rates ranging from 2.4 to 81% (Bambi et al. 2018), while a study of eldercare workers employed in both home care and nursing homes found a bullying prevalence of 7.7% (Hogh et al. 2018). While these estimates provide some indications of the occurrence of workplace aggression, there is still a shortage of studies on the prevalence rates in home care specifically.

Evidence from diverse occupational contexts show that exposure to aggression is associated with a range of outcomes on individual, organizational, and societal levels, which supports workplace aggression as a considerable psychosocial risk factor. Outcomes include, but are not limited to, mental distress (Hanson et al. 2015; Lanctôt and Guay 2014; Rudkjoebing et al. 2020; Verkuil et al. 2015), burnout (Hanson et al. 2015; Lanctôt and Guay 2014; Nielsen and Einarsen 2012; Rudkjoebing et al. 2020), PTSD (Lanctôt and Guay 2014; Nielsen et al. 2010), absenteeism and sick leave (Lanctôt and Guay 2014; Lee et al. 2023; Nielsen et al. 2016; Nyberg et al. 2021). Furthermore, the risk of sick leave after workplace violence has been found to be higher when the perpetrator is a colleague compared to a patient or client (Lee et al. 2023). In healthcare, this may stem from the belief that patient violence, in contrast to colleague perpetrated aggression, is “normalized” as part of the job (Bauersfeld and Majers 2023).

While few studies have examined workplace aggression in home care workers, the harmful effects should be equal to the impact on other healthcare workers. According to the Challenge-Hindrance-Threat Model (CHTM), exposure to workplace aggression represents a threat demand strongly associated with psychological distress and ill-health (Tuckey et al. 2015). Janoff-Bulman’s (1992) theory of shattered assumptions may explain this detrimental impact. The theory suggests that individuals rely on basic beliefs about the world, such as trust in others and self-worth, to maintain psychological stability. Workplace aggression disrupts these assumptions by violating the expectation of a safe, respectful work environment, leading to feelings of betrayal, helplessness, and anxiety. This disruption can cause a re-evaluation of one’s worldview, triggering psychological distress. Additionally, allostatic load theory (McEwen 1998, 2003) highlights how repeated stress from ongoing aggression or fear of recurrence can lead to cumulative physiological damage, increasing the risk of mental and physical illness, and contributing to clinical disorders and increased sickness absence.

In summary, there is a considerable knowledge gap in the literature regarding the prevalence and impact of workplace aggression among home care workers, particularly in Scandinavia. While violence against hospital and nursing home nurses has been well-studied, less attention has been given to home care workers, especially those with doctor-certified health outcomes (Nyberg et al. 2021). This study aims to fill this gap and support efforts to reduce mental distress and sick leave by testing the following hypotheses:

### H1

Employees exposed to physical and/or psychological workplace aggression will report significantly higher levels of clinically relevant symptoms of mental distress compared to those not exposed.

and

### H2

Exposure to physical and psychological workplace aggression is associated with an increased risk of sick leave due to common mental disorders.

## Methods

### Design and study sample

The present study utilized data from project aimed at evaluating the effect of the Norwegian Labour Inspection Authority’s regulatory tools (inspection and guidance) on the work environment and employee health (Indregard et al. 2019). Inspection focuses on a company's compliance with regulations for a safe working environment and health standards, including internal controls, health and safety, and labor laws such as wage rules for posted workers and proper staffing. Guidance refers to the Labour Inspection Authority’s advice regarding health, environment, and safety (HSE) regulations, working conditions, employment contracts, and staffing enterprises, with a primary focus on ensuring employers comply with the Norwegian Working environment act. The primary objective of the research project was to determine the impact of the Labour Inspection Authority’s regulatory tools on the psychosocial, organizational and mechanical work environment and employee health among Norwegian home care workers using a longitudinal, cluster-randomized, controlled trial. Randomization was conducted at the organizational level with municipalities as units. The examination of the regulatory tools was performed at the organizational level, whereas the outcome measures relating to the work environment factors and health complaints were assessed at the individual level. Outcome measures to evaluate compliance with OSH legislation were assessed at the group level. The findings of the trial showed that the Labour Inspection Authority’s regulatory tools had no significant impact on working conditions, subjective health outcomes, or sick leave (Garshol et al. 2022, 2023). The collected data was therefore considered as eligible for time-lagged analyses of relations between the study variables without adjusting for the impact of the regulatory tools.

**Fig. 1:**
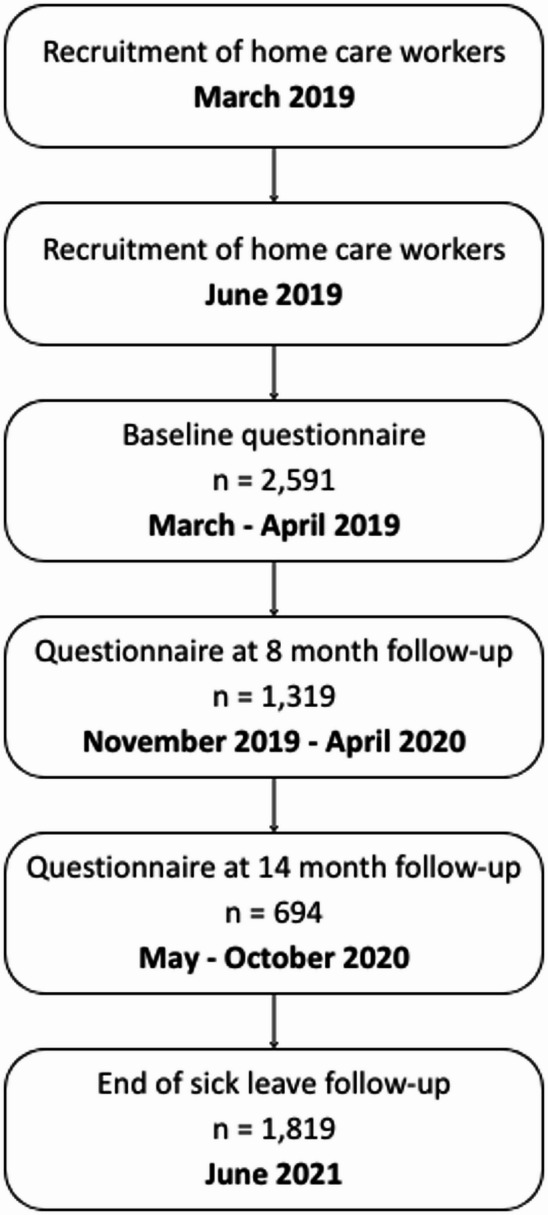
Recruitment and data collection process

Municipalities that received an inspection from the Labour Inspection Authority in 2017 or 2018 were not eligible for inclusion. A probability sample of 132 municipalities, whose home care service employed 20–100 workers, were invited to participate in March 2019. This initial range was chosen to reduce intra-cluster variability and included most of the eligible municipalities. An additional 48 municipalities employing 101–200 home care workers were invited in June 2019, as the range was expanded in order to increase the statistical power for subsequent studies of sick leave in the sector. A total of 180 municipalities were invited to participate, of which 130 (72%) accepted. All home care workers, which primarily included home care nurses (who provide professional medical care) and home care aides (who assist with personal care and housekeeping tasks), were invited via email to participate in three web-based surveys on working conditions and self-reported health outcomes. Questionnaire data on psychosocial work factors and subjective mental health outcomes were collected at baseline, with follow-up surveys conducted at 8- and 14-months post-baseline (see Fig. 1). Additionally, baseline survey data were linked to registry data on medically certified sick leave during a 26-month follow-up period. A total of 2591 of the 7103 invited employees responded at baseline, yielding an initial response rate of 36.5%. Furthermore, 1819 participants approved of collecting registry data on sick leave during the follow-up period, yielding a response rate of 25.6% for the sick leave analysis.

### Measures

#### Workplace aggression predictors

*Bullying by colleagues* and *supervisors* was measured using a formal definition emphasizing repeated negative acts and a power imbalance (Einarsen and Skogstad 1996), followed by two items asking whether participants had experienced bullying by colleagues or a supervisor in the past 6 months. Responses ranged from 1 (no) to 5 (yes, daily). *Physical violence and threats* were assessed using two items from Statistics Norway (Aagestad et al. 2014), asking whether participants had experienced violence or threats that caused fear in the past 12 months (or 6 months at follow-up), with response options from 1 (no) to 4 (weekly). For affirmative responses, participants indicated the perpetrator(s): supervisor, colleague, or patient/others. *Unwanted sexual attention* was measured with one item from Statistics Norway (Sterud and Hanvold 2021), asking whether participants had been exposed to such behavior at work. The same response scale and perpetrator identification were used as for violence and threats.

#### Mental distress

Symptoms of depression and anxiety were assessed using an abbreviated 5-item version of the Hopkins Symptoms Checklist (HSCL-5), which has been validated and demonstrates a robust correlation with the original 25-item scale (Tambs and Moum 1993). Participants were asked to rate how affected they had been by the following symptoms during the past 14 days: feeling fearful, nervousness or shakiness inside, feeling hopeless about the future, feeling blue, and worrying too much about things. Response alternatives were 1 = ‘not been affected at all, 2 = ‘not been affected much’, 3 = ‘been affected quite a lot’, and 4 = ‘been affected a great deal’. An index was computed using the mean of the item scores (α = 0.88 at baseline). Clinically relevant symptoms of mental distress were identified by a cut-off point of ≥ 2 on the HSCL-5 (Strand et al. 2003).

#### Sick leave

This study also utilized data on sick leave spells due to common mental disorders, as an extension of the self-reported mental distress measure. Registry data on sick leave was provided by the Norwegian Labour and Welfare Administration (NAV) for the 1819 participants who approved collection. The data constituted complete registrations of all medically certified sick leave compensated by the social insurance system, including its duration and diagnostic codes of the International Classification of Primary Care (ICPC-2) given by the general practitioner. Mental disorder-related sick leave was defined as medically certified sickness absence diagnosed within the ICPC category of psychological diagnoses (P) indicating anxiety, depression, and psychological complaints. The following ICPC-2 codes were included in the analysis: feeling anxious/nervous/tense (P01), anxiety disorder/anxiety state (P74), feeling depressed (P03), depressive disorder (P76), acute stress reaction (P02), psychological symptom/complaint/other (P29). The outcome was the number of sick leave spells for each participant due to the above-mentioned psychological diagnoses. We obtained sick leave data from March 2019 through June 2021, a follow-up period of 26 months. This data was linked to the baseline survey data through participants’ unique 11-digit national identity number.

### Covariates

Analyses were adjusted for self-reported *age, sex* (male or female), and *years of education* due to these variables’ established association with sick leave (Allebeck and Mastekaasa 2004). We also adjusted for the *percentage of full-time equivalent position* as the time spent at work and being exposed to work factors is likely to influence the risk of sick leave.

### Statistical analysis

All statistical analyses were performed using STATA 17.0 (StataCorp 2021), a comprehensive software package for data management, statistical analysis, and graphical representation commonly used in epidemiological and social science research. T-tests and chi squared tests were conducted to investigate differences in background characteristics, exposure levels, and outcomes at baseline between respondents at follow-up and respondents who dropped out after baseline. To determine whether workplace aggression impacts clinically relevant symptoms of mental distress (HSCL-5 score ≥ 2) we utilized mixed-effects logistic regression analysis with lagged effects. Mixed models are widely used for longitudinal data analysis due to their ability to handle dependencies in data with repeated measures as well as missing values. *Bullied by colleagues, bullied by supervisor, violence, threats of violence,* and *unwanted sexual attention* were entered into the model at t-1 (t1 and t2), and *mental distress* was entered at t (t2 and t3). Random intercepts were used to correct for clustering of variables within subjects. Separate models were fitted for each predictor, with adjustments for covariates. Odd ratios (OR) were calculated for the association between predictors and outcome. Mixed models generally handle dropout well, as they give unbiased estimates under the assumption that the data is missing at random (MAR). The MAR assumption implies that the mechanisms leading to dropout depend on the information in the collected data rather than on the values of the data that are missing (Fitzmaurice et al. 2011; Twisk et al. 2013). Remaining missing data on the study individual variables were handled by listwise deletion.

Negative binomial regression analysis was performed to investigate the relationship between workplace aggression and medically certified sick leave due to common mental disorders. Sick leave data is a form of count data that is often characterized by overdispersion, where the variance is larger than the mean (Christensen et al. 2007). It also tends to be zero-inflated, including more zero-values that indicate no absence from work. The negative binomial model is a generalization of the Poisson model, which has frequently been used to analyze sick leave data, that is better suited to handle overdispersion and zero-inflation than the Poisson model. We estimated incidence rate ratios (IRR) for the association between predictors and outcome measures, with robust standard errors that are robust against misspecification of the model and heteroskedasticity (unequal distribution of the error terms).

## Results

Characteristics of the main study sample, along with comparisons between respondents at follow-up and dropouts, are presented in Table 1. In the main sample (n = 2591), most participants were female (96%). The mean age was 45 years, 48% had 13 years of education or more, 36% worked full-time, and 86% spent half their work hours or more with clients. At baseline, the percentages of participants who reported experiencing one or more types of aggression during the last 12 months, were as follows: bullied by colleagues (13.2%), bullied by supervisor (8.1%), physical violence (12.8%), threats of violence (12.9%), unwanted sexual attention (27.0%). Violence, threats of violence, and unwanted sexual attention were mostly perpetrated by patients. Finally, 15.2% reported clinically relevant symptoms of mental distress (HSCL ≥ 2).

**Table 1:** Sample characteristics at baseline and baseline differences between respondents at follow-ups and dropouts

	Baseline sample n = 2591				Respondents at follow up n = 1426^a^				Dropouts at follow up n = 1113^b^				Sample differences^c^	
	n	%	Mean	SD	n	%	Mean	SD	n	%	Mean	SD	t-test	X^2^
Sex														*p* > 0.05
Male	128	4.9			64	4.5			61	5.5				
Female	2463	95.1			1362	95.5			1052	94.5				
Age			44.6	12.1			45.4	11.6			43.4	12.5	*p* < 0.05	
Education														*p* > 0.05
1– 9 years	84	3.4			39	2.9			43	4.0				
10– 12 years	1214	48.9			645	47.5			545	50.7				
13–16 years	1079	43.5			616	45.4			442	41.1				
> 16 years	106	4.3			58	4.3			45	4.2				
Missing	108				68				38					
Percentage of full-time equivalent employment			79.4	22.5			81.3	20.9			76.7	24.3	*p* < 0.05	
Aggression exposures														
Bullied by colleague	285	13.2			168	13.1			110	13.0			*p* > 0.05	
Missing	427				149				271					
Bullied by supervisor	174	8.1			99	7.7			70	8.3			*p* > 0.05	
Missing	429				149				273					
Physical violence	274	12.8			169	13.3			103	12.3			*p* > 0.05	
Missing	442				159				276					
Threats	277	12.9			169	13.3			105	12.5			*p* > 0.05	
Missing	440				157				276					
Sexual harassment	583	27.0			335	26.2			238	28.4			*p* > 0.05	
Missing	433				149				277					
Mental distress (range 1–4)			1.41	0.55			1.39	0.53			1.43	0.57	*p* > 0.05	
$\text{ HSCL}\ge$ 2	331	15.2			183	14.2			136	16.0			*p* > 0.05	
Missing	409				140				261					

^a^Respondents at baseline and at 8 months, 14 months, or both

^b^Respondents at baseline only

^c^Sample differences between respondents at follow-up and dropouts

There was significant attrition between surveys, with a 49.1% drop-out from baseline to T1 and 47.4% from t1 to t2. T-tests and chi-squared tests indicated no significant differences, regarding workplace aggression and mental distress, between respondents at follow-up and those who dropped out (Table 1). Dropouts at follow-up were on average slightly younger and had a lower mean percentage of full-time equivalent employment.

Characteristics of the subsample that accepted collection of sick leave data (n = 1819) are presented in Table 2. A total of 773 participants (42.5%) experienced sick leave due to any cause during the 26-month follow-up period, and of these, 146 participants (18%) experienced sick leave due to common mental disorders. T-tests were conducted to examine differences between the participants who did and those who did not consent to collection of sick leave data on mean scores of all exposures, covariates, and self-reported health measures. The results indicated no major differences between the two groups (table not shown).

**Table 2:** Characteristics of the sick leave subsample

Variables	Total		No medically certified sick leave		Medically certified sick leave due to common mental disorders	
	n = 1819	(%)	n	(Cases %^1^)	n	(Cases %^2^)
*Age*						
< 30	242	13.3	139	57.4	21	8.7
30–39	342	18.8	192	56.1	39	11.4
40–49	477	26.2	270	56.6	50	10.5
50–59	520	28.6	297	57.1	31	6.0
> 59	238	13.1	148	62.2	5	2.1
*Sex*						
Female	1734	95.3	987	56.9	142	8.2
Male	85	4.7	59	69.4	4	4.7
*Work status*						
Permanent employment	1716	94.9	983	57.3	138	8.4
Temporary contract	38	2.1	23	60.5	3	7.9
Substitute/extra	52	2.9	33	63.5	5	9.6
Other	3	0.2	1	33.3	–	–
Missing	10					
*Working with clients*						
No contact	10	0.6	8	80.0	–	–
Less than half the time	260	14.3	167	64.2	19	7.3
Half the time or more	1532	84.2	862	56.3	127	8.3
Missing	17					
*Aggression exposures*						
Bullied by colleagues	223	12.3	192	86.1	31	13.9
Missing	5					
Bullied by supervisor	140	7.7	122	87.1	18	12.7
Missing	6					
Physical violence	229	12.7	208	90.8	20	8.7
Missing	16					
Threats of violence	227	12.6	206	90.8	21	9.3
Missing	14					
Unwanted sexual attention	483	26.7	440	91.1	43	8.9
Missing	7					

^1^ Percentage within each category with no medically certified sick leave

^2^ Percentage within each category with medically certified sick leave due to common mental disorders

Chi-square tests of independence were performed to determine whether the proportion of each exposure was equal between sexes at baseline (table not shown). Results showed no sex differences in exposure to bullying, violence, and threats. However, the difference between the percentage of female workers who reported experiencing unwanted sexual attention (27.7%) and the percentage of male workers (12.6%) was statistically significant, ×^2^(3, N = 2157) = 10.88, *p* = 0.013.

The prospective associations between workplace aggression and clinically relevant symptoms of mental distress are presented in Table 3. Results suggest that exposure to all five types of workplace aggression, *bullied by colleagues* (OR 3.96, 95% CI 2.95–5.30), *bullied by supervisor* (OR 3.64, 95% CI 2.60–5.10), *violence* (OR 1.70, 95% CI 1.25–2.31)*, threats of violence* (OR 2.40, 95% CI 1.72–3.35)*,* and *unwanted sexual attention* (OR 1.73, 95% CI 1.39–2.16), were significantly associated with higher odds of mental distress. Thus, H_1_ was supported.

**Table 3:** Odds ratios of workplace aggression on clinically relevant mental distress

	Crude		Adjusted^a^	
	OR	95% CI	OR	95% CI
Bullied by colleagues	3.91	(2.94–5.20)	3.96*	(2.95–5.30)
Bullied by supervisor	3.49	(2.52–4.84)	3.64*	(2.60–5.10)
Physical violence	1.75	(1.29–2.37)	1.70*	(1.25–2.31)
Threats of violence	2.36	(1.71–3.27)	2.40*	(1.72–3.35)
Unwanted sexual attention	1.86	(1.50–2.31)	1.73*	(1.39–2.16)

^a^Adjusted for age, sex, education, and percentage of full-time equivalent position

^*^p ≤ 0.001

The prospective associations between workplace aggression at baseline and sick leave due to common mental disorders during follow-up are presented in Table 4. In the adjusted model, only *bullied by colleagues* (IRR 1.62, 95% CI 1.17–2.23) was significantly associated with an excess risk of sick leave. Hence, H_2_ was partially supported.

**Table 4:** Incidence risk ratios for sick leave due to common mental disorders

	Crude		Adjusted^a^	
	IRR	95% CI	IRR	95% CI
Bullied by colleagues	1.58	(1.17–2.14)	1.62*	(1.17–2.23)
Bullied by supervisor	1.33	(0.96–1.85)	1.28	(0.91–1.80)
Physical violence	0.88	(0.63–1.23)	0.88	(0.63–1.23)
Threats of violence	1.32	(0.79–2.21)	1.29	(0.70–2.38)
Unwanted sexual attention	1.07	(0.85–1.34)	0.96	(0.75–1.23)

^a^Adjusted for age, sex, education, and percentage of full-time equivalent position

^*^p ≤ 0.005

## Discussion

This prospective study examined the prevalence of physical and psychological workplace aggression among home care workers and its impact on mental distress and certified sick leave due to common mental disorders. Based on theory and previous research, we hypothesized that workplace aggression toward home care workers would be prospectively associated with 1) symptoms of mental distress, and 2) an excess risk of sick leave due to common mental disorders.

A total of 13.2% of home care workers reported being bullied by colleagues, 8.1% reported being bullied by supervisors, 12.8% experienced physical violence, 12.9% experienced threats of violence, and 27.0% experienced unwanted sexual attention. The majority of violence, threats, and unwanted sexual attention were perpetrated by patients or others not employed in the home care services. The prevalence rates established in this study are higher than in the general working population in Norway. In a nationwide study it was found that 3.3% experienced bullying by colleague or leader, 5.0% experienced physical violence, 8.8% experienced threats of violence, and 4.7% experienced unwanted sexual attention (Statistics Norway 2023). However, the abovementioned study also showed that the home care services are one of the sectors with the highest rates of workplace aggression, particularly regarding unwanted sexual attention, where more than one in four employees are exposed. Taken together, our findings support these results by showing that home care workers have a high risk of exposure to workplace violence. Exposure to bullying was also highly prevalent in our study. This is in line with a recent review emphasizing that bullying may be more prevalent in the healthcare context (Goh et al. 2022). Risk factors for bullying are for example found to include types of work and gender ratio at the workplace (Ortega et al. 2009; Salin et al. 2013), such as female-dominated occupations working with clients or patients—conditions that are typically found in healthcare. Characteristics of the work that are prominent in the home care sector, such as work overload, staff shortages, and stressful working conditions (Andersen and Westgaard 2013), are previously found to be antecedents of bullying among nurses (Karatuna et al. 2020).

Research on sex differences in workplace aggression shows mixed results, with prevalence often varying by occupation (Salin et al. 2013). While some healthcare studies report significant sex differences in exposure to violence, reviews generally find no consistent patterns. (Guay et al. 2014, 2015). Similarly, this study found no significant sex differences in exposure to bullying, physical violence, or threats. However, male workers in healthcare, where they are often a minority, have been shown to face higher risks of physical violence and bullying. Notably, we found a significant sex difference in exposure to unwanted sexual attention, with 27.7% of women affected compared to 12.6% of men. This aligns with prior findings that women are disproportionately targeted in workplace sexual harassment (McDonald 2011).

Hypothesis 1 was fully supported as all indicators of aggression were associated with higher levels of mental distress. This finding is in line with most previous research on aggression and mental health outcomes (Hanson et al. 2015; Lanctôt and Guay 2014; Rudkjoebing et al. 2020; Verkuil et al. 2015). Hypothesis 2 was partially supported, as only *being bullied by colleagues* was associated with an increased risk of sick leave due to common mental disorders. This finding is in line with results from systematic reviews suggesting a consistent association between workplace bullying and sick leave (Lever et al. 2019; Nielsen et al. 2016; Nyberg et al. 2021). The associations between exposure to supervisor-perpetrated bullying, physical violence, threats of violence, and unwanted sexual attention were not significant. In contrast to our findings, Clausen et al. (2012) reported that violence and threats increased the risk of long-term sick leave among eldercare workers. Similarly, Aagestad et al. (2014) found these factors to be strong predictors of long-term sick leave among female health and social workers. However, a review by Nyberg et al. (2021) concluded that while some Nordic studies suggest a link between physical violence and sick leave in this sector, the overall evidence remains too limited to confirm a consistent association.

Several studies using a general working population sample have found significant associations between forms of physical and psychological violence and non-specific sick leave (Friis et al. 2018; Hoffmann et al. 2020; Niedhammer et al. 2008, 2012; Slany et al. 2014). However, these studies often do not distinguish between types of perpetrators, and perceptions of aggression may vary by occupation. Research that separates colleague and patient/client aggression shows that victimization by colleagues or supervisors has more severe consequences, such as higher risks of sick leave (Lee et al. 2023), post-traumatic reactions (Geoffrion et al. 2018), reduced job satisfaction and everyday functioning, and symptoms of burnout (Merecz et al. 2009). In our study, the types of aggression not linked to sick leave (physical violence, threats, and unwanted sexual attention) were mostly perpetrated by patients. Reactions to workplace aggression often depend on the perpetrator's role (Merecz et al. 2009), shaping how the incident is experienced. Patient aggression is typically seen as an organizational issue, often met with support, whereas colleague-perpetrated bullying can lead to social isolation and reduced support.

Healthcare workers often normalize patient aggression by perceiving it as an unfortunate but accepted part of the job (Grasmo et al. 2021; Nakaishi et al. 2013). This normalization suggests that such aggression might be seen as a challenge necessary to overcome in order to continue performing caregiving tasks (Zhang et al. 2021). Being bullied by colleagues or supervisors is unlikely to be rationalized in the same way and should therefore be perceived as more uncontrollable and distressing. Finally, the outcomes of different types of aggression can be influenced by power dynamics. Power, defined as the capacity to influence others and produce intended effects (Dunbar and Bernhold 2019), plays a significant role in these interactions. In the employee-patient relationship, the worker generally holds more power and has greater control over the situation. However, when subjected to bullying, the target is inherently powerless, as the power imbalance prevents them from stopping the mistreatment. As a result, victims of bullying may experience feelings of resignation or helplessness. Hence, in line with the CHTM, bullying may be appraised as more of a threat than patient aggression within the healthcare context, which may explain why only the association between being bullied by colleagues and sick leave reached statistical significance.

### Practical implications

Our findings highlight workplace aggression as prevalent exposure with potential adverse effects for the mental health and sickness absence rates among home care workers. Interventions to reduce and prevent aggression, and especially bullying, may contribute to maintain good mental health, and thereby also reducing the levels of sick leave. Organizations should prioritize primary, secondary, and tertiary interventions combined in an integrated approach (Nielsen et al. 2023; Rugulies et al. 2023). Reviews of interventions for violence in the health and social sector have highlighted that while individual level training programs may positively impact on workers’ perceived ability to deal with aggressive behavior, reduction in violence rates likely require multicomponent interventions (Geoffrion et al. 2020; Somani et al. 2021).

In terms of primary prevention, cultivating a strong psychosocial safety climate (PSC) within organizations appears to be one of the most effective strategies for mitigating workplace aggression (Law et al. 2011). Building such a climate requires active and visible commitment from senior management, including: (1) demonstrating support for psychological health through consistent involvement and taking prompt, decisive action to address psychosocial risks; (2) prioritizing employee well-being above productivity pressures; (3) maintaining transparent communication with employees regarding issues that may impact psychological health and safety; and (4) engaging stakeholders, including employees, unions, and health and safety representatives, in occupational health and safety processes through meaningful participation and consultation (Nielsen et al. 2023). As for secondary prevention strategies, well-developed reporting systems in combination with strong “ethical infrastructure” that enables a climate for constructive conflict management have been found to be highly important with regard to managing cases of aggression, and of workplace bullying in particular (Einarsen et al. 2017, 2018). Additionally, supervisor support seems to be beneficial after being exposed to aggression (Lamothe et al. 2021). Finally, treatment programs for tertiary prevention should be highly prioritized. Such programs must aim to reduce the negative effects of violence, threats, and bullying, as well as rebuilding the victim’s trust and security in their colleagues, patients, and the organization (Nielsen et al. 2023).

### Strengths and limitations

This study’s main strengths include its prospective design, probability sampling, and the use of registry-based, diagnosis-specific sick leave data, in line with Nyberg et al. (2021). The sampling strategy supports external validity, making the results largely generalizable to other public home care services in Norway and to healthcare workers more broadly. However, generalizability to other countries may be limited by differences in sick leave policies and compensation schemes.

All data on workplace aggression and mental distress came from self-report surveys, raising potential for common method variance and reporting biases (Podsakoff et al. 2003; Spector 2006). These were mitigated through measures such as separating variables in the questionnaire, the time-lagged design, and ensuring anonymity, and using varied response formats. The response rate of 36.5% may seem low but aligns with typical averages for survey-based studies (Stedman et al. 2019). Importantly, prior research suggests that response rates have limited influence on the internal validity of findings (Beehr et al. 2022; Hendra and Hill 2019; Phillips et al. 2016). Therefore, the obtained response rate is unlikely to significantly affect the observed relationships between variables in this study. Substantial attrition occurred over time, likely due to high turnover in the sector. However, attrition analyses showed no significant differences in workplace aggression or mental distress between follow-up participants and dropouts. While a healthy worker effect is a potential concern (Li and Sung 1999), prior research among Norwegian workers suggests it may not meaningfully bias findings in this context (Nielsen and Knardahl 2016).

Using medically certified registry data minimized attrition and reporting bias. Common method bias was avoided by sourcing predictors and outcomes from different datasets (Podsakoff et al. 2003). Robust standard errors addressed heteroscedasticity and multiple testing, making results conservative and increasing risk of Type II errors. Still, the association between bullying and sick leave due to mental disorders is unlikely to reflect a Type I error. The design does not allow for conclusions about whether mental health issues increase the risk of bullying. While meta-analyses show both directions of influence (Theorell et al. 2015; Verkuil et al. 2015), reverse effects tend to disappear in studies with longer time lags (Nielsen and Einarsen 2018). Also, the lack of data on prior mental disorders limits conclusions about the onset vs. recurrence of sick leave.

Workplace aggression was assessed via self-report, which may introduce underreporting—particularly for patient-initiated behaviors, due to normalization in healthcare settings (Bauersfeld and Majers 2023; Zhong and Shorey 2023). Prevalence estimates vary depending on measurement method (Nielsen et al. 2010; Nyberg et al. 2021). This study used the self-labeling approach, where respondents identify and report experiences via single-item measures. For bullying, a definition accompanied the item. Compared to behavior-based checklists, this approach may underestimate exposure (Parveen et al. 2023). A meta-analysis on workplace aggression found that methodology, sampling, and geography contribute to variability in reported prevalence rates (Nielsen et al. 2010).

## Conclusion

This study contributes to understanding workplace aggression in home care and its associations with mental health and sick leave. All forms of aggression were linked to increased risk of mental distress, but only colleague-perpetrated bullying showed a clear association with sick leave due to common mental disorders. Preventive efforts should prioritize reducing workplace bullying, while also addressing the effects of patient aggression. Future research should explore strategies to prevent all forms of aggression in home care and identify factors that may buffer its mental health impacts. Additionally, combining self-labeling and single-item measures could help clarify which incidents workers experience, how they perceive them, and their relation to health outcomes (Nyberg et al. 2021).

## Data Availability

The data that support the findings of this study are available from the Norwegian Agency for Shared Services in Education and Research (SIKT), but restrictions apply to their availability. The data were used under license for the current study and are not publicly available. Any data access requests must be submitted to SIKT for review (https://sikt.no/en/home).
